# Effects of Raloxifene Combined with Low-dose Conjugated Estrogen on the Endometrium in Menopausal Women at High Risk for Breast Cancer

**DOI:** 10.6061/clinics/2021/e2380

**Published:** 2021-01-11

**Authors:** Andrea Lucia Bastos Carneiro, Ana Paula Curi Spadella, Fabiola Amaral de Souza, Karen Borelli Ferreira Alves, Joaquim Teodoro de Araujo-Neto, Mauro Abi Haidar, Rita de Cássia de Maio Dardes

**Affiliations:** Departamento de Ginecologia, Escola Paulista de Medicina (EPM), Universidade Federal de Sao Paulo (UNIFESP), Sao Paulo, SP, BR

**Keywords:** SERMs, Endometrium, Estrogen, Raloxifene

## Abstract

**OBJECTIVES:**

To compare the effects of low-dose conjugated estrogen (CE), raloxifene, and the combination thereof on the endometrium of postmenopausal women.

**METHODS:**

Postmenopausal women between 45 and 60 years of age, with Gail score≥1.67 and no endometrial disorders, were randomly assigned to receive low-dose CE (0.3 mg), raloxifene (60 mg), or combined therapy for 1 year. Transvaginal ultrasound was performed at baseline and every 3 months; the Kupperman Index was assessed at baseline and every 6 months. Endometrial biopsies were performed if endometrial thickness (ET) was ≥5 mm or if vaginal bleeding occurred. The primary outcome was the occurrence of ET≥5 mm over the one-year period.

**RESULTS:**

Seventy-three women were randomly assigned and analyzed on an intent-to-treat basis. Eight, three, and four women in the CE, raloxifene, and combination groups, respectively, exhibited ET≥5 mm. No genital bleeding was reported in the combination group. Endometrial biopsy revealed atrophy or polyps in all groups, with one patient in the CE group exhibiting a proliferative endometrium without atypia. At 6 months, there was a progressive increase in mean ET in the CE group, but not in the other two groups, with statistically significant differences at 6, 9, and 12 months. Mean scores for vasomotor symptoms and Kupperman Index favored the CE and combination groups over raloxifene.

**CONCLUSION:**

Combined raloxifene and low-dose CE decreased the severity of menopausal symptoms to a similar extent as CE alone and had similar effects as raloxifene alone on the endometrium.

## INTRODUCTION

The management of menopausal symptoms remains controversial. In the past decade, therapies other than estrogen plus progestogen have been increasingly studied (1), and it has become accepted that successful treatment depends on individual assessment of a woman’s needs and the consequent analysis of the risk-benefit profile. Among the selective estrogen receptor modulators (SERMs) and compounds with varying affinities for estrogen receptors in different target tissues, raloxifene acts as an estrogen agonist on the bone and serum-lipid metabolism, and as an estrogen antagonist in the breast and uterus (2-8). Raloxifene is currently approved for the prevention and treatment of postmenopausal osteoporosis and chemoprevention of invasive breast cancer (9). In postmenopausal women with osteoporosis, raloxifene has been reported to decrease the risk for breast cancer by 66% (10). However, despite the favorable profile, including endometrial safety, raloxifene may lead to increased occurrence of hot flashes and, as such, may not be well-tolerated.

Conjugated estrogens (CEs) have been confirmed to reduce hot flashes and are among the compounds that remain considered to be optimal for the treatment of vasomotor symptoms (1). Hormone replacement therapy is often associated with a good benefit-risk profile in women <60 years of age and within 10 years of onset of menopause (1). However, unopposed estrogens may induce endometrial proliferation in women with an intact uterus (1). Low-dose estrogens, despite inducing less endometrial proliferation than standard doses (11,12), are administered in combination with progestogens to ensure long-term endometrial safety.

Because women undergoing estrogen plus progestogen therapy have been shown to have an increased risk for breast cancer (13), in contrast to those treated with unopposed estrogen who appear to have a reduced risk (14), in addition to the concerns regarding an increased risk for cardiovascular events raised by the Women’s Health Initiative study, alternative options for hormone replacement therapy can be considered. Tissue-selective estrogen complex, which comprises the combination of estrogen with a SERM, has been explored in attempts to optimize hormone replacement therapy and reduce adverse outcomes (15), including endometrial proliferation. Combinations of various estrogen formulations and raloxifene have been described both *in vitro* and *in vivo* (16-24), and have yielded controversial results regarding cell proliferation patterns in the endometrium, bone, and breast tissues. The combination of CE and raloxifene could potentially minimize the undesirable effects of estrogen on the uterus and breast tissues, and allow the beneficial agonistic effects of estrogen in other estrogen target-tissues (25-29).

The present study was part of a larger research project that aimed to compare low-dose CE alone *versus* raloxifene alone or a combination thereof in postmenopausal women at high risk for breast cancer. This specific population may experience increased endometrial reactivity and, could therefore, experience different outcomes in terms of endometrial proliferation when exposed to raloxifene combined with estrogens (30). The present study aimed to compare low-dose CE alone *versus* raloxifene alone or a combination thereof in terms of endometrial thickness (ET) and histology, genital bleeding patterns, and menopausal symptoms among postmenopausal women at high risk for breast cancer.

## METHODS

### Study design and oversight

The present investigation was a double-blind, randomized trial conducted at the Federal University of São Paulo, São Paulo, Brazil. Postmenopausal women at high risk for breast cancer, as assessed according to the Gail algorithm, were randomly assigned to one of three groups: low-dose CE (0.3 mg), raloxifene (60 mg), or combined therapy (*i.e.*, CE+raloxifene), all for 1 year. To maintain masking, each of the three treatments was provided in one capsule identical in appearance to the other two treatments, with only the study pharmacist being aware of treatment assignment. The study medication was provided to patients every 3 months during follow-up visits. Women were instructed to take one capsule per day for 1 year. The study protocol was approved by the Institutional Review Board (Research Ethics Committee of the Federal University of São Paulo-Paulista Medical School (UNIFESP/EPM) under number (1766/08), and adhered to the principles of the Helsinki Declaration. All women provided written informed consent before participation in the trial.

### Patient eligibility and assessment

Healthy women with a normal uterus were eligible if they were between 45 and 60 years of age, experienced amenorrhea for at least 12 months, a serum estradiol level ≤20 pg/mL, and a Gail score≥l.67. Women with uterine bleeding of unknown etiology, significant pelvic pathology as determined by screening Pap-smear, gynecological examination, and transvaginal ultrasonography, irregular ET or ET≥5 mm as determined by transvaginal ultrasonography, a uterine cavity that could not be evaluated at baseline, a history of estrogen-dependent cancer or thromboembolic disease, those who used corticosteroids, estrogen, or a progestogen within 6 months before study commencement, and those with chronic diseases that were not well controlled, were excluded from the study.

The Kupperman Index (KI) was assessed at baseline, and at 6 and 12 months. Vasomotor symptoms were characterized as absent, mild, moderate, or severe using scores 0, 1, 2, or 3, respectively.

Transvaginal ultrasound was performed at baseline and every 3 months. The KI was assessed at baseline and every 6 months, and vasomotor symptoms were characterized as absent, mild, moderate, or severe using scores 0, 1, 2, or 3, respectively. Hysteroscopy and endometrial histological evaluations were performed if ET≥5 mm or genital bleeding was reported. Patients in whom ET≥5 mm was detected by transvaginal ultrasound at any time point were discontinued from the study treatment but continued with follow-up until the planned study completion at 1 year.

### Outcomes of interest

The primary outcome was the presence ET≥5 mm at any time point. Secondary outcomes included the variation in ET over the one-year treatment, vaginal bleeding, the severity of menopausal symptoms, and KI.

### Statistical analysis

The sample size for the present trial was computed based on the primary objectives of the larger research project, which addressed issues related to the breast. Therefore, there was no sample-size calculation for the current endometrial substudy, which included all patients randomized in the trial following the intent-to-treat principle. Potential differences between groups were assessed both overall (across the three groups) and in pairwise comparisons. For overall comparisons, the Fisher’s exact test or one-way analysis of variance were used for categorical and numerical variables, respectively. For pairwise comparisons, the Fisher’s exact test was used for categorical variables and *t* tests for independent samples were used for numerical variables. The occurrence of either an ET≥5 mm or vaginal bleeding was assessed as a composite endpoint using Kaplan-Meier analysis, with comparison across the three groups using the log-rank test. Cox regression models were used to assess the potential influence of age, body mass index (BMI), time since menopause, parity, and Gail index for the risk of occurrence of such events. The relative risk (RR) of ET≥5 mm with corresponding 95% confidence interval (CI) was calculated using the raloxifene group as reference. Differences with *p*<0.05 were considered to be statistically significant.

## RESULTS

### Patient characteristics

A total of 73 women were randomly assigned to one of the three groups (*i.e.*, low-dose CE [0.3 mg]); raloxifene [60 mg]; or combined therapy). The numbers of patients completing the 1-year follow-up were 19 in the CE, 18 in the raloxifene, and 24 in combination groups, respectively. Ten early discontinuations were due to loss to follow-up, and two were due to consensual withdrawal. As shown in Table 1, the three groups were balanced for several baseline characteristics, including age, age at menopause, time since last period, Gail index, BMI, and ET. At baseline, the mean ET values were 2.28 mm, 2.15 mm, and 2.05 mm, for the CE, raloxifene, and combination therapy groups, respectively.

### Primary outcome

**Figure 1:**
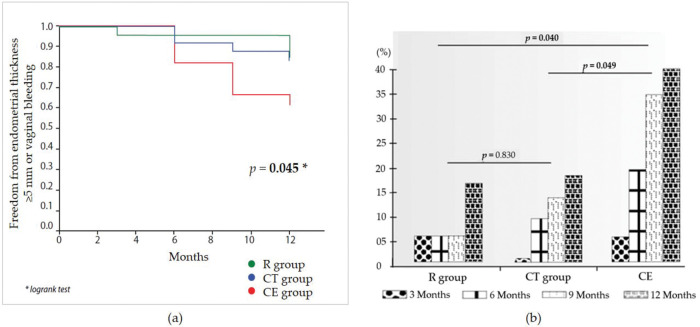
**a)** Kaplan-Meier analysis of the occurrence of either an endometrial thickness of ≥5 mm or vaginal bleeding over the 1-year study period. **b)** Risk assessment of endometrial thickness increase >5 mm throughout the study by study group. R: raloxifene; CT: combined therapy (low-dose conjugated estrogen plus raloxifene); CE: conjugated estrogen.

Fifteen women exhibited ET≥5 mm throughout the study (eight, three, and four women, in the CE, raloxifene, and combination groups, respectively). Compared with the raloxifene group, there was a non-significant increase in the risk for ET≥5 mm in the CE group (RR 3.00 [95% CI 0.79-11.29]; *p*=0.072). There was no statistically significant difference between the raloxifene and combination groups (RR 1.13 [95% CI 0.25-5.05]; *p*=0.457). Genital bleeding was reported once for each of the three women in the CE group. These three patients exhibited ET>5 mm at the first scheduled ultrasound assessment after the bleeding (one at 6 months and two at 12 months). Women in the raloxifene and combination groups reported no genital bleeding. Figure 1a illustrates the occurrence of either ET of ≥5 mm or vaginal bleeding over the 1-year study period, with a statistically significant difference across the study arms. Although no pairwise comparisons were performed, the incidence of these outcomes was nominally higher in the CE group than in the other two groups. The odds of increased ET>5 mm during 1 year of treatment are shown in Figure 1b. The raloxifene group was not different from the combination therapy group (*p*=0.830), while women in the CE group exhibited an increased risk for endometrial thickening during the study relative to the raloxifene and combination therapy groups (*p*=0.040 and *p*=0.049, respectively).

Among the 15 women who exhibited ET≥5 mm, 11 underwent evaluation of endometrial samples, while four were lost to follow-up (three from the CE group and one from the raloxifene group). All endometrial biopsy results from women allocated to the raloxifene and combination groups revealed atrophy or endometrial polyps. In the CE group, atrophy and endometrial polyps were also observed, except in one woman who was diagnosed with proliferative endometrium without atypia at 9 months of treatment.

### Secondary outcomes

**Figure 2:**
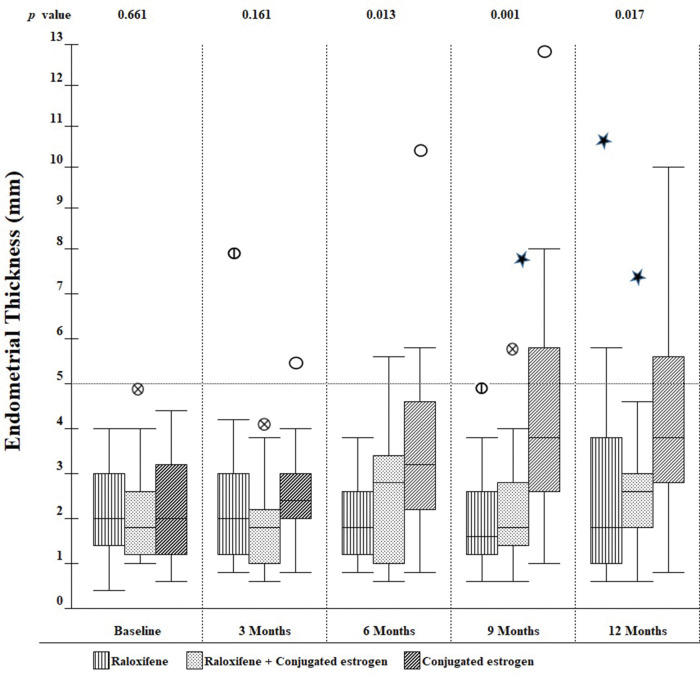
Endometrial thickness in the three groups over the 1-year period of observation.

Over the one-year observation period, differences in ET emerged across the three groups. As shown in Figure 2, at 6 months, there was a progressive increase in mean ET among those in the CE group, but not in the raloxifene or combination groups. As a result, the differences across the three groups were statistically significant at the 6-, 9-, and 12-month visits.

Regarding the severity of vasomotor symptoms, the mean (±standard deviation) baseline scores were 1.15±1.05, 1.45±1.06, and 1.48±1.12 for the CE, raloxifene, and combination groups, respectively (*p*=0.492). At 6 months, the corresponding values were 0.32±0.65, 1.00±0.89, and 0.28±0.61, with statistically significant differences across groups (*p*=0.002), and for the pairwise comparisons between both the CE (*p*=0.003) and combination (*p*=0.001) groups and the raloxifene group. At 12 months, the mean scores were 0.35±0.59, 1.11±1.05, and 0.35±0.65; once again, we observed statistically significant differences across the groups (*p*=0.003), and for the pairwise comparisons between both the CE (*p*=0.004) and combination (*p=*0.003) groups and the raloxifene group.

There was an overall reduction in KI during the study in the three groups, with the only statistically significant difference being observed at 6 months when both the CE (3.8±4.02) and combination (4.2±5.77) groups demonstrated mean values lower than the raloxifene group (8.7±7.58; *p*=0.014 for the comparison across groups). At 12 months, nominal differences persisted between the three groups (5.2±5.66, 4.8±6.13, and 8.6±7.57, respectively); however, the difference across the three groups was not statistically significant (*p*=0.133).

## DISCUSSION

We studied the effect of raloxifene plus low-dose CE on menopausal symptoms and the endometrium in postmenopausal women at high risk for breast cancer. We found promising results in terms of decreased severity of vasomotor symptoms and endometrial findings. An increase of >5 mm in ET was observed in four women in the combination group, assessed as atrophy (n=2) and endometrial polyp (n=2) at endometrial biopsy. No statistical difference was found between endometrial findings for the raloxifene and combined therapy groups.

Raloxifene therapy has not been implicated in an increased risk for endometrial dysplastic or neoplastic disorders (5,31). Safety data from two large trials (6,10) demonstrated that raloxifene was associated with an increased rate of endometrial polyps (3.2% *vs* 1.9% placebo; *p*=0.028) but not of endometrial hyperplasia or uterine cancer when compared with placebo (*p*≥0.66). On the other hand, raloxifene therapy is associated with increased vasomotor symptoms, which may limit the success of treatment.

The combination of raloxifene with estrogens have been described both *in vitro* and *in vivo* (16-24). The antagonistic effect of different SERMs (raloxifene, tamoxifen, bazedoxifene, and lasofoxifene) on estradiol-treated endometrial epithelial Ishikawa cells has been demonstrated by altered expression of different genes (*HOXA10, LIF, PR,* and *EMX2*) (16). The expression of *EMX2*, a gene involved in suppression of the proliferation of endometrial cells, was not significantly increased in endometrial cells exposed to raloxifene alone or combined with estrogen (16), which potentially explains the occurrence of endometrial hyperplasia in some clinical trials (29), which was not observed in the raloxifene plus estrogen group in our study. In another *in vitro* study using Ishikawa cells and the non-malignant immortalized human endometrial glandular cell line EM1, it was noted that tamoxifen or raloxifene associated with 17-beta estradiol had little effect on estrogen metabolites, estrogen-DNA adduct formation, or the expression of estrogen-metabolizing enzymes in endometrial cells (24). Although *in vitro* studies of different SERMs associated with estrogens are available, including the evaluation of raloxifene and estrogens, clinical trials with these compounds remain scarce.

Clinical trials investigating raloxifene plus estrogens have used heterogeneous methodologies, with variability in the type of estrogen used and route of administration (32). Additionally, many studies have focused on the transition from hormonal therapy to raloxifene rather than on long-term therapy. In the current study, we used the KI to evaluate the severity of vasomotor symptoms, which is consistent with the study by Valiati et al. (26), but different from other studies investigating raloxifene plus estrogens (25,27-29). An improvement in KI was shown in the combination group at 6 months, alongside a significant decrease in the severity of vasomotor symptoms at 6 and 12 months. Overall, our results are consistent with most previous studies involving combined estrogens and raloxifene, which reported benefits in menopausal symptoms.

The average ET at baseline in our study across the three groups was 2.16 mm, with no difference among the groups (*p*=0.661). The mean ET was not increased in the combination group when compared with baseline values or with the raloxifene group during the study. These results are consistent with other studies, which also did not identify significantly increased ET when raloxifene was combined with estrogens (26-28). On the other hand, two other studies (25,29) reported increased ET with the use of raloxifene combined with esterified CEs (0.312 mg/day) or 17-beta-estradiol (1 mg/day). The difference between these results and those of our study may be due to the different type of estrogen used and/or route of administration. Moreover, in the former study (25), there was a numerically higher average ET in the pre-treatment phase, and the latter (29) had statistically significant differences in average ET at 52 weeks when compared with baseline and raloxifene group, with two patients developing endometrial hyperplasia.

Genital bleeding, a commonly reported event in women who use estrogen, is related to dose and exposure time (33). However, the use of low-dose estrogen (equivalent to 0.3 mg of CEs) dramatically reduces the incidence of genital bleeding (11,33). Although often considered a result of cell proliferation, genital bleeding is also commonly observed in women with endometrial atrophy (34). This may explain the low incidence of this complaint in low-dose estrogen users, as reported in a two-year study (11). In the present study, endometrial analysis was performed every 3 months to ensure that eventual early changes in ET would be promptly recognized and treated accordingly. Among the population studied, three women using low-dose CE alone experienced genital bleeding, and all three exhibited ET >5 mm on the first assessment by ultrasound after the bleeding. Of these, two were diagnosed with endometrial atrophy and one exhibited simple proliferative endometrium without atypia. Although four women in the combination group were noted to have ET >5 mm in our study, neither genital bleeding nor endometrium hyperplasia occurred in this group. Most clinical trials that assessed the combined use of raloxifene plus estrogens did not report the occurrence of genital bleeding (26-28).

The present study had a few limitations, the first of which was its small sample size. Moreover, some women included in this study had a high BMI, which, although homogeneous among the three groups, may have influenced the results. Obesity itself may cause increased ET because it contributes to the production of estrogens by adipose tissue. Investigating the correlation between endometrial thickening and obesity or a high BMI was beyond the scope of this trial and not feasible with this sample size. Strengths of the present study include the originality of the use of the combination of raloxifene and estrogen in postmenopausal women at high risk for breast cancer and quarterly assessment of the endometrium during the one-year study period. Additionally, the lack of published studies investigating marketed drug associations to treat common menopause in women makes this study clinically important.

Results of the present one-year study demonstrate the clinical similarity of endometrial findings between menopausal women receiving raloxifene alone and those receiving raloxifene plus low-dose CEs. These results are clinically significant and add to previously published studies that evaluated the combination of raloxifene and estrogens. To our knowledge, this is the first study to evaluate the effects of combined therapy using combined CEs in postmenopausal women at high risk for breast cancer. This specific patient population currently lacks a treatment option that is both safe and effective to both reduce the risk for cancer and to maintain good quality of life. We believe that larger studies investigating the safety of this association in postmenopausal women at high risk for breast cancer are warranted.

## CONCLUSION

This one-year trial demonstrated that combined raloxifene and low-dose CEs decreased the severity of menopausal symptoms to an extent similar to CE alone, and had effects similar to raloxifene alone on the endometrium.

## AUTHOR CONTRIBUTIONS

Carneiro ALB, Souza FA, Alves KBF, Araujo-Neto JT, Dardes RCM collected the clinical data. Dardes RCM was involved in the conceptualization. Dardes RCM and Haidar MA supervised the work. Dardes RCM and Carneiro ALB were involved in the planning, analysis, and interpretation of data. Carneiro ALB and Dardes RCM drafted the manuscript. Spadella APC designed the figures. All authors discussed the results and approved the manuscript final version to be submitted for publication.

## Figures and Tables

**Table 1:** Patient demographic information at baseline.

	Group			
Variable	R (n=22)	CT (n=25)	CE (n=26)	*p*
Age, years	56.7±3.76	56.9±4.86	56.5±3.29	0.915
Gail index	2.4±0.88	2.0±0.29	2.1±0.49	0.101
Age at first period, years	13.4±2.04	13.9±1.98	12.7±1.55	0.056
Age at menopause, years	49.0±3.42	48.9±4.46	49.3±3.77	0.902
Time since last period, years	7.9±4.68	7.6±5.81	7.5±4.37	0.964
Endometrial thickness, mm	2.15±0.85	2.05±0.93	2.28±0.99	0.661
First-degree relative with breast cancer	9 (40.9)	14 (56)	16 (61.5)	0.681
Body mass index, kg/m^2^				
<24.9	1 (4.5)	0 (0)	1 (3.8)	0.730
25-29.9	10 (45.5)	11 (44)	8 (30.8)	
30.0-39.9	11 (50)	14 (56)	17 (65.4)	

Data presented as mean±standard deviation or n (%), unless otherwise indicated. R: raloxifene; CT: combined therapy (conjugated estrogen with low-dose plus raloxifene); CE: conjugated estrogen
